# Author's Reply: Positive models of suffering and psychiatry

**DOI:** 10.1192/bjb.2024.28

**Published:** 2024-06

**Authors:** Ahmed Samei Huda

**Affiliations:** Consultant Psychiatrist, Pennine Care NHS Foundation Trust, UK. Email: ahmed.huda@nhs.net

*Beginnings of a dialogue within our profession*

I thank Professor Prakash and Professor Breen for their helpful and informed contributions. It is important in the spirit of medicine, which is a profession that is used to working with different professions and different models of care, that we engage with the positive model of suffering if that is the wish of the patient. Professor Prakash outlines a model of how to do so as a medical practitioner. Professor Breen supplements this with both how we can incorporate the positive model in our care and suggestions for working with others who are more experienced in the positive model of suffering. I hope this article stimulates further helpful suggestions and contributions.

